# Flavones’ and Flavonols’ Antiradical Structure–Activity Relationship—A Quantum Chemical Study

**DOI:** 10.3390/antiox9060461

**Published:** 2020-05-27

**Authors:** Maciej Spiegel, Tadeusz Andruniów, Zbigniew Sroka

**Affiliations:** 1 Department of Pharmacognosy and Herbal Medicine, Wroclaw Medical University, Borowska 211A, 50-556 Wroclaw, Poland; zbigniew.sroka@umed.wroc.pl; 2 Advanced Materials Engineering and Modelling Group, Department of Chemistry, Wroclaw University of Science and Technology, M. Smoluchowskiego 23, 50-372 Wroclaw, Poland; tadeusz.andruniow@pwr.edu.pl

**Keywords:** flavonoids, polyphenols, antioxidants, quantum chemistry, density functional theory (DFT), structure–activity relationship

## Abstract

Flavonoids are known for their antiradical capacity, and this ability is strongly structure-dependent. In this research, the activity of flavones and flavonols in a water solvent was studied with the density functional theory methods. These included examination of flavonoids’ molecular and radical structures with natural bonding orbitals analysis, spin density analysis and frontier molecular orbitals theory. Calculations of determinants were performed: specific, for the three possible mechanisms of action—hydrogen atom transfer (HAT), electron transfer–proton transfer (ETPT) and sequential proton loss electron transfer (SPLET); and the unspecific—reorganization enthalpy (RE) and hydrogen abstraction enthalpy (HAE). Intramolecular hydrogen bonding, catechol moiety activity and the probability of electron density swap between rings were all established. Hydrogen bonding seems to be much more important than the conjugation effect, because some structures tends to form more intramolecular hydrogen bonds instead of being completely planar. The very first hydrogen abstraction mechanism in a water solvent is SPLET, and the most privileged abstraction site, indicated by HAE, can be associated with the C3 hydroxyl group of flavonols and C4’ hydroxyl group of flavones. For the catechol moiety, an intramolecular reorganization to an o-benzoquinone-like structure occurs, and the ETPT is favored as the second abstraction mechanism.

## 1. Introduction

The twofold nature of reactive oxygen and nitrogen species (ROS, RNS) in the organism is broadly reported [1,2,3]. They not only participate in signal transduction [3,4], but also may lead to the breaking of DNA chains [5], lipid peroxidation [6] and protein decomposition [7]. During oxidative stress, the free radical concentration overwhelms natural antioxidants’ capacity, damaging cells and initiating severe diseases such as atherosclerosis [8], neoplasms [9] and Parkinson’s [10] or Alzheimer’s [11] disease.

Flavonols and flavones belong to a large group of polyphenolic compounds of flavonoids, known for their beneficial activity, deriving from the antiradical potential [12]. With a capacity to scavenge free radicals and a wide distribution in vegetables [13], they play a crucial role as an external source of antioxidants. Therefore, it is important to maintain their recommended intake.

Their antioxidative ability was found to depend greatly on the molecular structure and substitution pattern: availability of hydroxyl groups—their absolute and relative position, as well as their number [14,15,16,17]; the stabilizing effect of hydrogen bonds—intramolecular and originating from the solvent [18,19]; the electron delocalization across a molecule, which considerably relies on the degree of conjugation [16,20] and hyperconjugation effect [21]; and the substituents effect, especially of methoxy groups [22].

It is believed that A-ring substituents are not directly involved into the scavenging mechanism [17,23]. Therefore, the type of the B-ring substitution is considered as a determinant of flavonoids’ antiradical potency—even one hydroxyl group located there guarantees noticeable scavenging potential, especially if it is in position C4’. Similar observations were noted for phenolic acids [16,24,25]. Furthermore, highly active flavonoids usually also possess a catechol moiety the activity of which, found in other classes of polyphenolic compounds, was demonstrated recently [15,26,27]. The C2–C3 double bond extends π-conjugation onto the carbonyl group in the C-ring, so the unsaturated flavonoids’ radical scavenging ability is greater than in saturated structures, e.g., flavanones [28,29,30]. Herein, the catechol moiety is a subject of the investigation in this manuscript as, according to several reports [17,31,32], it can play a significant role in scavenging potential.

Appropriate assessment of flavonoids’ activity requires in-depth studies on all possible modes of action: hydrogen atom transfer (HAT), electron transfer–proton transfer (ETPT), and sequential proton loss–electron transfer (SPLET) [33,34]; as well as their defining characteristics, and the mathematical values related to them: bond dissociation enthalpy (BDE)—for HAT; ionization potential (IP) and proton dissociation enthalpy (PDE)—for ETPT; proton affinity (PA) and electron transfer enthalpy (ETE)—for SPLET. Mechanism-independent determinants, reorganization enthalpy (RE) and hydrogen abstraction enthalpy (HAE) were calculated as well, but for more general, kinetic-independent purpose [35].

This study was focused on describing the structure–activity relationship (SAR) and determinants of the flavones’ and flavonols’ activity, with robust, computational chemistry methods. The investigations include 13 flavonoids differing in a substitution pattern (Table 1). An intramolecular swap reaction was discovered and examined for the catechol moiety in a thermodynamic aspect, and the relevance of the hydrogen bonding in the B-ring was evaluated and the electronic structure deeply explored, involving the spin density distribution, chemical hardness, as well as HOMOs and LUMOs analysis. The obtained results provide the basis for understanding flavonols’ and flavones’ antioxidative potential and explain the differences between them.

## 2. Materials and Methods

All energies noted as kcal/mol were converted from atomic units (a.u.) according to the conversion factor, where 1 a.u. equals 627.5 kcal/mol.

### 2.1. Conformer Geometry Generation

Molecules of the studied flavonoids were generated in Avogadro [36] from their simplified molecular-input line-entry system (SMILES) [37]. The obtained structures were used in the Gabedit10 [38] Amber Molecular Dynamics Conformational Search procedure to obtain the lowest energy conformers. 1.0 ps of heating followed by 1.0 ps of equilibration molecular dynamic protocols were employed. After completion, obtained conformational isomers of each flavonoid were used for quantum chemistry studies.

Each structure was first optimized in a vacuum with Gaussian16 [39] using the HF/3-21G(d) model chemistry method and then with density functional theory (DFT) B3LYP/6-31G(d,p) [40]. Very tight geometry optimization cutoff and an ultrafine integration grid were used for vibrational frequency calculations. All flavones exhibited exactly one imaginary frequency, indicating that a planar conformation is a first-order saddle point on the potential energy surface. For this reason, the C2–C1’ bond of each conformer was successively rotated by 60 degrees and saved for further elaboration, until reaching total 300 degrees of rotation from the origin. Obtained geometries were recalculated at the same computational chemistry level of theory as earlier and all real frequency values were confirmed. The lowest energy conformers were selected as the representatives and optimized once more, but in a polarizable continuum model (PCM) of water solvent, using B3LYP/6-31G + G(d,p) method [41,42]. Calculated enthalpies of the lowest energetical flavonoid isomers are shown in Table S1.

It is interesting to point out that based on our results the gas phase equilibrium structures of flavonols are flat, with the notable exception of morin, while flavones reveal strained geometries, in accord with other studies [43]. The planarity of flavonols is lost in water environment contradictory to Todorova et al.’s [44] findings. In fact, according to our B3LYP/6-31+G(d,p) calculations (this trend was also noticed in a larger basis set, such as 6-31+G(d,p) or aug-cc-pVDZ), all flavonols but morin possess a very flat potential energy surface (PES) for dihedral angles, describing the distortion from planarity, spanning a region of PES ranging from 0 degrees to its equilibrium structure value seen in Table 2. The energy difference between the flat and strained compounds is tiny, up to 0.10 kcal/mol, however, the strained structure always has the lower energy and all real vibrational frequencies, in contrast to the one imaginary frequency found for planar structures of galangin, fisetin and myrecetin. It is worth noting that these conclusions hold true for re-optimized equilibrium geometries with an unpruned grid (Grid = 199974) and very tight optimization criteria, as well as re-calculated vibrational frequencies for such high-quality geometries.

### 2.2. Radical Geometry Generation

The representative geometries from the previous step served as an input for radical calculations. This step includes removing a single hydrogen atom from each hydroxyl group and running computations at UB3LYP/6-31+G(d,p), retaining an implicit water solvent in two ways: with and without geometry optimization. Spin contamination values of open shell DFT results were checked, as they may interfere with the outcome [45,46]. All were in a range of <0.7500, 0.7511> after spin annihilation. Cation-radical, anion-radical and triplet diradical calculations used later in this study were elaborated the same way. The outcomes obtained for the first two forms were in the same range as for radicals, whilst for the latter one they were found in a range of <2.0001, 2.0030>. The ideal values, calculated according to the formula *s*(*s* + 1) where *s* is a half of a number of unpaired electrons, are 0.75 for a radical and 2.0 for a triplet diradical. Since DFT results are in the acceptable range, they could be used for the elaboration. Calculated enthalpies are presented in Table S2. Structural parameters of flavonoids, such as dihedral angles around the C2–C1’ bond (θ) and corresponding values for relaxed radicals (θ^●^), are shown in Table 2.

### 2.3. Quantitative Determinants of Antioxidant Potential

The enthalpy values, *H(x),* used in this section refer to the unrelaxed forms, include thermal correction and were obtained following Hessian calculations by employing Gaussian16 software [39]. Reorganization enthalpy is the only one that makes use of relaxed radicals’ results. The values of enthalpy for H^+^, H^●^ and e^−^ in a water solvent were taken from another study [47].

#### 2.3.1. Hydrogen Atom Transfer Mechanism

The hydrogen atom transfer is the simplest reaction path an antioxidant can undergo and is based on a homolytic bond dissociation between the hydrogen and the oxygen atom in the hydroxyl residue:

$$ArOH_{relaxed}\to ArO_{unrelaxed}^{●}+H^{●}$$

A quantitative descriptor of this process can be assigned to the bond dissociation enthalpy [BDE; Equation (1)], defined as the change of the enthalpy after the hydrogen abstraction [Equation (2); see results in Table 3]:

(1) $$BDE=H\left(ArO_{unrelaxed}^{●}\right)+H\left(H^{●}\right)-H\left(ArOH_{relaxed}\right)$$

(2) HAT=BDE

#### 2.3.2. Electron Transfer–Proton Transfer Mechanism

Another recently proposed mechanism is a two-step sequence of electron release from the molecule, followed by a proton dissociation from the formed cation-radical:

$$ArOH_{relaxed}\to ArOH_{unrelaxed}^{●+}+e^{-}$$

$$ArOH_{unrelaxed}^{●+}\to ArO_{unrelaxed}^{●}+H^{+}$$

The enthalpy of this process is the sum of the adiabatic ionization potential [IP; Equation (3)] and the proton dissociation enthalpy [PDE; Equation (4)], which can be calculated as follows [Equation (5); see results in Table 4]:

(3) $$IP=H\left(ArOH_{unrelaxed}^{●+}\right)+H\left(e^{-}\right)-H\left(ArOH_{relaxed}\right)$$

(4) $$PDE=H\left(ArO_{unrelaxed}^{●}\right)+H\left(H{\;}^{+}\right)-H\left(ArOH_{unrelaxed}^{●+}\right)$$

(5) ETPT=IP+PDE

#### 2.3.3. Sequential Proton Loss–Electron Transfer Mechanism

This path consists of a proton dissociation from the investigated compound and an emission of the free electron afterwards:

$$ArOH_{relaxed}\to ArO_{unrelaxed}^{-}+H^{+}$$

$$ArO_{unrelaxed}^{-}\to ArO_{unrelaxed}^{●}+e^{-}$$

The whole reaction is the proton affinity [PA; Equation (6)] enthalpy plus the electron transfer enthalpy [ETE; Equation (7)] [Equation (8); results are presented in Table 5]:

(6) $$PA=H\left(ArO_{unrelaxed}^{-}\right)+H\left(H^{+}\right)-H\left(ArOH_{relaxed}\right)$$

(7) $$ETE=H\left(ArO_{unrelaxed}^{●}\right)+H\left(e^{-}\right)-H\left(ArO_{unrelaxed}^{-}\right)$$

(8) SPLET=PA+ETE

#### 2.3.4. Mechanism-Independent Determinants

The non-specific indices—the reorganization enthalpy [RE; Equation (9)] and the hydrogen abstraction enthalpy [HAE; Equation (10)]—were also used for a quantitative analysis of the flavonols’ and flavones’ antioxidative potential.

The reorganization enthalpy describes the energy change upon shift from the unrelaxed to the relaxed form, hence geometry optimization. Thus, it may be an interesting predictor of conformational changes resulting from the hydrogen abstraction:

$$ArO_{unrelaxed}^{●}\to ArO_{relaxed}^{●}\;$$

(9) $$RE=H\left(ArO_{relaxed}^{●}\right)-H\left(ArO_{unrelaxed}^{●}\right)$$

The hydrogen abstraction enthalpy is independent of the scavenging mechanism and its kinetics. It serves as a general determinant of the flavonoid activity that includes reorganization enthalpy correction [35]:

$$ArOH_{relaxed}\to ArO_{relaxed}^{●}+H^{●}\;$$

(10) $$HAE=H\left(ArO_{relaxed}^{●}\right)+H\left(H^{●}\right)-H\left(ArOH_{relaxed}\right)$$

The obtained RE and HAE values are presented in Table 6.

## 3. Results and Discussion

### 3.1. Molecule and Radical Electronic Structure Investigation

The starting point of the backbone examination was undertaken with a natural population analysis of NBO 3.1 [48] software, implemented in Gaussian16 [39]. In view of the fact that the backbone is identical for flavones and flavonols, the following statements can be ascribed to both of them, with some exceptions indicated in the text.

Natural bonding orbitals (NBO) analysis revealed that every C–C bond is composed of two *sp^2^* orbitals, so each carbon atom also has one unoccupied *p_y_* orbital. This can lead to the assumption that free electrons, located on oxygen atoms connected with the aromatic ring, will likely interact with orbitals of carbon atoms, in conjugation and hyperconjugation effects.

Thanks to the *p_y_* orbitals’ conjugation, the system’s total energy is lowered. This property is highly dependent on the AC- and B-rings’ mutual planarity, and as discussed above, the B-ring does not share the same plane as the AC-complex. The dihedral angle varies (Table 2) depending on intramolecular repulsions or hydrogen bonding caused by C3, C2’ or C5’ residues. Dimitrić Marković et al. [27], explaining the differences in activity between anthocyanidins, delphinidin and pelargonidin against C3-glycosylated anthocyanin, malvin, suggested that the greater activity of the first two is due to the C3 hydroxyl group, which maintains coplanarity of the B- and C-ring, and hence *p_y_* orbitals’ conjugation. Nevertheless, in this study something completely different was observed. Flavonols reach exact planar structure when hydrogen from the C3 hydroxyl group is removed. Moreover, considering reorganization enthalpy as a descriptor of the most favorable abstraction site in terms of geometry change, it is indeed the C3 hydroxyl group. It can be concluded that, upon reaction, the torsion caused by this residue does not have an impact on the B-ring anymore, and rotation is thermodynamically favored. When HAE is investigated, C3 residues are generally favored, unless the investigated flavonol has at least two hydroxyl groups nearby. Then, one of their positions is an abstraction site. That way it can be expected that hydrogen bond stabilization energy is more urgent than *p_y_* orbital conjugation. Contrarily, for all flavonols but morin, abstraction from C4’ also leads to the planarity of the rings. This can be associated with the formation of radical which, to be transferred onto the C-ring, require double bond formation between the p_y_ orbitals of C2 and C1’.

The hyperconjugation effect was investigated considering molecular orbitals (MOs) interactions. One can see in Figure S1 that the oxygen atoms’ LUMO phases have a different sign than aromatic carbon atoms, indicating possible interaction. Indeed, Milenković et al. [21], in their study on kaempferol structure, found how greatly the interference of oxygen atoms’ free electrons with the antibonding orbital of carbon atoms contributes to structure stabilization, decreasing the system’s total energy by up to 34 kcal/mol. However, the structure as a whole is not conjugated. The existence of the carbonyl group in the C-ring, due to the cross-conjugation effect, divides the compound into two electron-separated ring complexes—AC and BC [49]. Therefore, electron density cannot flow freely, in a strict orbital manner, from the A-ring to the B-ring or in reverse, as was stated by Jovanović et al. [50]. To check the other possibility, a hydrogen shift between C5 and the carbonyl residue was elaborated (Appendix A) and its results also go against this hypothesis. Even more, this postulate can be refuted with examination of radicals’ spin density distribution—upon forming a radical in the A or B-ring, the density is nearly 0 in B and A-ring atoms, respectively (Figure S2).

The C2–C3 saturation limits electrons’ delocalization, indirectly decreasing reduction potential. Evidence confirming this thesis can be found by studying the activity results’ comparison between flavonols and flavones against flavanols [51] or flavanones [52] in simple assays, e.g., ferric ion-reducing antioxidant power (FRAP) [53], 2,2’-azino-bis (3-ethylbenothiazoline-6-sulfonic acid) diammonium salt (ABTS) [54], and 2,2-diphenyl-1-picrylhydrazyl (DPPH) [55] or isoflavones in an acrylamide reduction test [56].

**Figure 1:**
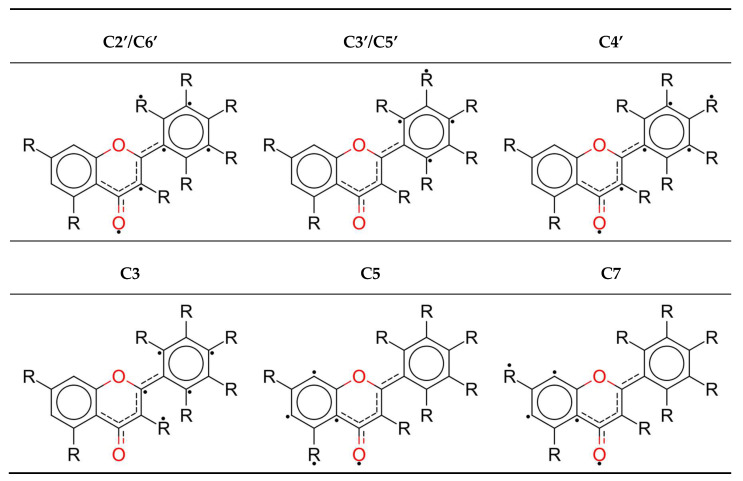
Lewis structures of radicals with the biggest electron densities of atoms marked with a dot.

Removal of the hydrogen atom by an abstractor creates a radical form, for which Lewis resonance structures are shown in Figure 1. One can see that the biggest resonance effect occurs for the C2’, C6’ and C4’ radicals; it is lower for the C3, C5 and C7 radicals, and the lowest for the C3’ and C5’ radicals.

Formerly presented HAE values (Table 6) are accepted in this study as a main numerical determinant of antioxidative potential, as they are mechanism-independent. In compliance with them, preferred hydrogen abstraction position patterns can be depicted. Just based on HAE values, flavonoids, indeed, can be divided into two groups: (I) compounds with a C3 hydroxyl group (flavonols); (II) compounds lacking a hydroxyl residue at the C3 position (flavones).

For flavones, the C4’ position, if present, is the most active one. The reason for that may be attributed to larger delocalization of the electron density upon radical formation, and the possibility of establishing the hydrogen bond with the adjacent hydroxyl residues at C3’ or C5’. Similar observations were derived from studies on the activity of phenolic acids [57] or anthocyanidins [27], where C4’ had the most favorable hydrogen abstraction energy. Elaboration of this process was performed and is demonstrated in a latter section (Section 3.3), where the importance of intramolecular hydrogen bonds is confirmed. The A-ring hydroxyl residues have much higher HAE, up to 25 kcal/mol, so their scavenging potential will be lower; thus, these in the B-ring seem to be the main determinants of antioxidant capacity, as was mentioned in the Introduction section.

However, flavonols with an OH group at C3 and a lone hydroxyl moiety at C4’ are more likely to convey hydrogen from the first position instead of the latter one, when HAE is compared, e.g., morin and kaempferol. On the other hand, if there are at least two (e.g., quercetin) or even three (e.g., myricetin), the C4’ position will be privileged. This indicates that the *p_y_* conjugation is desired only when the radical formed at the B-ring is not stabilized by at least one intramolecular hydrogen bonding.

### 3.2. Morin

**Figure 2:**
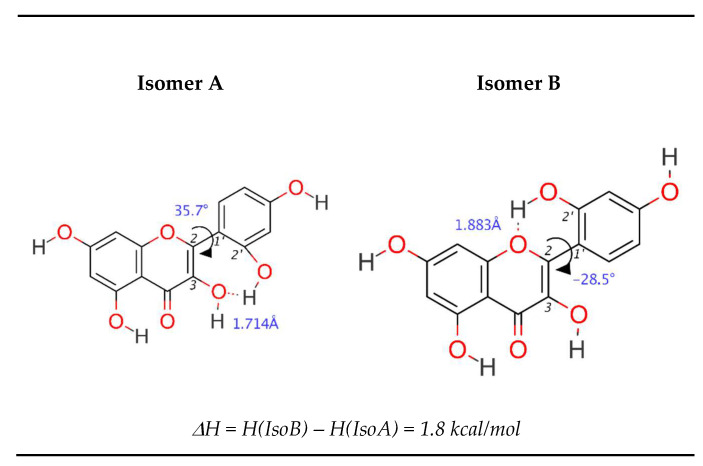
Comparison in geometry between morin’s A and B isomer.

Interesting properties, noted by Amić et al. [25], are exhibited by morin (Figure 2). In this study, it was found that the preferred molecular isomer is not the more planar one (isomer B), but the one able to form a hydrogen bond between C3–C2’ residues (isomer A). The difference in enthalpies between these conformers is ~1.8 kcal/mol, and this value seems to be sufficient to break the tendency to planarity. One can name previously noted steric restrictions disabling complete conjugation of *p_y_* orbitals, but the choice of isomer A can also be explained differently—the C3–C2’ hydrogen bond is “retained” even after the radical is formed, from either the C2’ or C3 hydroxyl group. In the first case, the hydrogen atom from the C3 hydroxyl group rotates from the carbonyl site to the B-ring site, forming a hydrogen bond with the C2’ radical, subsequently forcing rotation up to 45° due to the steric effect. In the second case, when the C3 radical is formed, it is not necessary for the hydrogen atom to move, and only C2–C1’ bond rotation is performed. This situation is most likely to occur since no energy is used for hydrogen shift, and planarity is achieved.

### 3.3. Mechanisms of Action in Terms of the Determinants

The very first step of the mechanism is considered as a thermodynamic determinant of a favored pathway.

#### 3.3.1. Hydrogen Atom Transfer Mechanism

The only investigated mathematical descriptor associated with the HAT mechanism is the bond dissociation enthalpy (Table 3). The lowest is associated with forming the C4’ radical, whilst C3’ and C3 enthalpies are quite similar. A likely explanation of this could be Mulliken spin density (SD) distribution in the created radicals (Figure S2). The lower the spin density at the radical center, the greater is the delocalization, and the formed radical becomes more stabilized with resonance. For all compounds but morin, the lowest spin density is associated with the C4’ radical. However, delocalization cannot be clearly stated as the only determinant of BDE—if it was, the lowest BDE value for fisetin should be at C4’ (SD = 0.284), whilst it is C3’ (SD = 0.316); for morin, the lowest BDE should be for C2’ (SD = 0.303), whilst it is C4’ (SD = 0.311). Therefore, it is assumed that some other factors may be crucial to describe the HAT mechanism. One can observe that C4’ BDE decreases with the number of adjacent hydroxyl groups—the lowest enthalpy was found for myricetin, where two hydroxyl groups surround C4’, while significantly higher values were derived for quercetin, luteolin and fisetin, with only one adjacent hydroxyl group. Conversely, compounds where the C4’ hydroxyl group is alone exhibit the highest bond dissociation enthalpies. Thus, it can be stated the HAT definitely depends on intramolecular hydrogen bonding.

#### 3.3.2. Electron Transfer–Proton Transfer Mechanism

The IP values are much bigger than BDE, while PDE is relatively low (Table 4). Since the first step determines the thermodynamically favored reaction pathway, the ETPT mechanism is not likely to be responsible for flavonoid activity. Nevertheless, ionization potential was also examined with frontier molecular orbitals theory (Appendix B).

The lowest energy required for ionization is 108.9 kcal/mol for fisetin and quercetin, while the highest is over 120 kcal/mol for chrysin; these are much greater values than any bond dissociation energies for the same compounds. Even though PDE does not show any regular pattern in the lowest energy centers, it definitely shows the highest—PDE values for the C5 position are the largest. The reason for this may be found in an electronegative repulsion between the carbonyl oxygen and the C5 radical’s unpaired electron (the distance between these atoms is about 2.9Å), as well as breakage of the hydrogen bond between these two residues.

#### 3.3.3. Sequential Proton Loss–Electron Transfer Mechanism

The SPLET mechanism is determined with proton affinity. Calculations showed that PA values are much lower than the corresponding values for the reaction path determinants of the HAT (BDE) or ETPT (IP) mechanisms. Therefore, this one appears to represent the most favored mechanism of action for flavonoids in a water (polar) solvent, as could be expected according to the other studies [27,58], including qualitative structure–activity relationship (QSAR) analysis [59,60,61]. This is rather surprising as, in general, A-ring hydroxyl groups are considered not to possess scavenging potential [17,62], whilst abstraction takes place at the C7 position for most of the investigated compounds. Gibb’s free energies of deprotonation, reported by Álvarez-Diduk et al. [63], are in agreement with the obtained results: the first or second pK is linked with hydrogen dissociation from the C7 hydroxyl group. Interestingly, Lin et al. [24], measuring activity of flavonoids with the DPPH assay, noted that upon glycosylation of C7 in luteolin, its antiradical activity noticeably decreased. They attributed this effect to the decreased availability of the free hydroxyl groups. Whilst the lowest proton affinity enthalpy of luteolin is assigned to C4’ (27.4 kcal/mol), the second lowest is assigned to C7 (31.3 kcal/mol). An explanation that could be proposed is the activity of a catechol moiety and a change of polarity within the structure, resulting in overall decreased activity of the compound, instead of a strict change of a favored location. Moreover, since SPLET’s ETE determinant is adequate for ETPT’s IP, and is lower than this, it can be assumed that an electron transfer is preferred for an ion form instead of a molecule. Based on the discussion conducted earlier for the ETPT mechanism, similar reasons can explain why the highest PA values are found for C5.

#### 3.3.4. Mechanism-Independent Determinants

The performed elaborations considered unrelaxed forms of intermediate compounds. The proposed reorganization enthalpy is a mathematical explanation of a conformer relaxation, decreasing the total enthalpy. Therefore, one can calculate reorganization enthalpy and, based on derived values, as well as chemical structure, identify significant geometry changes. For example, the lowest RE is for the morin C3 hydroxyl group, which was described earlier in the context of hydrogen bonds and planarity. The low value of RE for C5 can be justified by an electronegative interaction between the C5’ radical and carbonyl oxygen atom, which is mediated by a molecule by changing its geometry.

Although three mechanisms and their determinants were investigated, no reaction path can be proposed. It greatly depends on the solvent [34,64] and the abstractor molecule [65]. The lowest enthalpy only indicates where the abstraction is most likely to occur, not how often it will happen. For this reason, a hydrogen abstraction enthalpy can be used, and was used for further elaboration. It points out the most active compound or group, without taking into account how the radical state was reached, rejecting kinetic studies but involving reorganization of geometry. Herein, C4’ seems to be the most prominent one, and similar conclusions were stated by Dimitrić Marković et al. [64,66] and Sroka et al. [35]. Furthermore, the second most favored position in a SPLET mechanism is also C4’, so the importance of reorganization enthalpy correction may be bigger than one would expect.

#### 3.3.5. Antioxidant Capacity Summary

Assuming an activity is inversely proportional to the enthalpy required for the first step of the mechanism to occur, the investigated compounds have been sorted in the descending order (Table 7). As one can see, flavonols are more active than flavones, and structures with a greater number of hydroxyl groups stand out as the most active ones. The pattern of activity does not change greatly, apart from for flavone luteolin, which is in the top three when HAT and SPLET mechanisms are examined, overtaking most of the flavonols.

### 3.4. Intramolecular Hydrogen Bonding

The polyhydroxy structure of flavonoids provides an opportunity to form an intramolecular hydrogen bond if at least two hydroxyl groups are close to each other. Hydrogen bonds are known for increasing stability of both the molecule and the radical, hence decreasing the energy required to form the radical [19]. Such a situation can actually be found for every flavonol, where the C3 or C5 hydroxyl group interacts with a carbonyl residue or B-ring hydroxyl groups [21].

Fisetin, luteolin and quercetin do possess two hydroxyl moieties, while myricetin has three at the B-ring. This allows intramolecular hydrogen bonds to be formed when hydrogen is abstracted. Generally, the C4’ hydroxyl group can interact with either C5’ or C3’, whilst C5’ and C3’ can interact only with C4’. The exception among this group is morin, since its C3 group can interact with C2’, leading to a twisted structure. Moreover, a hydrogen bond can be formed between carbonyl group and C3 and C5 residues as well. This study focused only on the hydrogen bonding in B-ring groups, since C4’ (or C3’ for fisetin) was indicated as a favored position in a thermodynamically preferred SPLET mechanism, as well as as a mechanism-independent HAE determinant. In order to measure a hydrogen bonding stabilizing effect on a radical molecule, the difference in the enthalpies between the radical without (*ArO_NHB_*) and with the hydrogen bonds (*ArO_HB_*) was considered, named here as hydrogen bond enthalpy (*HBE*), and ascribed to the following equation:

(11) $$HBE=H\left(ArO_{NHB}^{●}\right)-H\left(ArO_{HB}^{●}\right)$$

**Figure 3:**
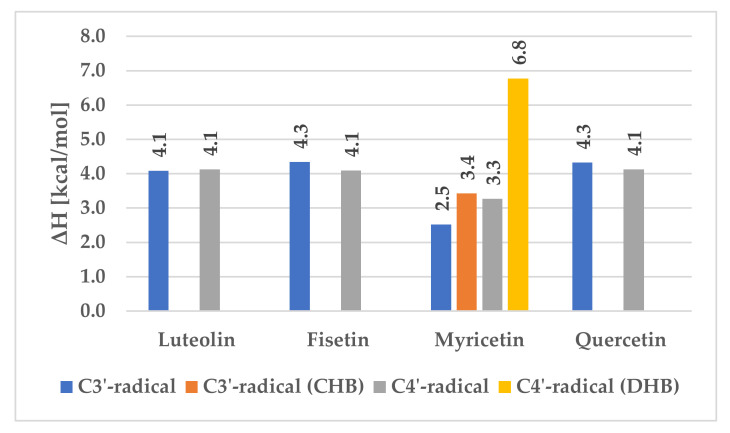
Differences in hydrogen bond enthalpy.

Since myricetin possesses three hydroxyl groups in the B-ring, in close proximity, two more situations had to be considered. For the C3’ radical, the C5’ hydroxyl group can be facing the same direction as C4’—named here as a cross hydrogen bond (CHB)—or the opposite one. On the other hand, the C4’ radical can be stabilized by two, one or zero hydrogen bonds coming from the C3’ and C5’ hydroxyl moieties. This leads to the three different situations, as presented in Figure 3, where C4’-radical (DHB) stands for the situation when the hydrogen bond is formed with both C3’ and C5’ hydroxyl hydrogens. The C3’ and C4’ radicals assume existence of only one hydrogen bond with a near-situated hydroxyl group.

The results are presented below, while the full list of enthalpies is appended to the Supplementary Materials (Figure S3):

For all compounds except myricetin, one can see that the average value of the hydrogen bond stabilization energy is about 4 kcal/mol, regardless of the radical site. For myricetin, C3’ stabilization energy without crossed hydrogen bond is lower by 1.5 kcal/mol than any other C3’ HBE, because oxygen electronegative repulsion happens over a short distance (2.718Å), decreasing hydrogen bond stability. However, if all hydrogen bonds are present, this energy difference increases to 3.4 kcal/mol, still being lower than for fisetin, luteolin or quercetin. For the C4’ radical, the stabilization energy is lower than the average, because electrons of at least one oxygen interact with the radical at C4’ (2.702Å). If there are two hydrogen bonds, the stabilization energy is much greater, reaching up to 7 kcal/mol.

### 3.5. Catechol Moiety

**Figure 4:**
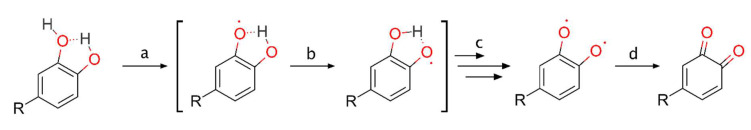
Example of catechol moiety hydrogen swap reaction and reorganization mechanism for C3’.

Sroka et al. [35] observed an interesting behavior of luteolin, in comparison to structurally similar apigenin. The only difference between them is the existence of a 3,4-diOH catechol moiety in luteolin, and this small dissimilarity resulted in a nearly 100 times greater activity of the compound. Formation of a diradical and its rearrangement to 1,2-benzoquinone is widely stated as an explanation for the antioxidative scavenging potential difference between these already-mentioned structures. Lin et al. [24], in their experimental study, found a large difference of activity between the C3’,C4’-dihydroxyl moiety flavonoids and those with a single C4’ residue. They have proposed a mechanism explaining this diversity (see Figure 4): (a) a hydrogen abstraction from the most favored position, herein indicated by the HAE value; (b) a hydrogen transfer from the C4’ to the C3’, if allowed; (c) a second hydrogen abstraction from the C3’ hydroxyl group; (d) an intramolecular reorganization and the hydroquinone formation. Within this section, it was investigated in a strict thermochemical scope.

#### 3.5.1. Intramolecular Hydrogen Swap

For all possible B-ring radicals, a synchronous transit-guided quasi-Newton method [67] was conducted to search potential energy surface for imaginary frequencies, corresponding to the presented movement of the hydrogen atom (b). Each compound demonstrated exactly one imaginary value. Next, the optimization to a transition state showed that it is similar to the dioxolane, where the hydrogen atom is suspended between oxygen atoms.

To describe these processes in a thermochemical way, Gibb’s free energy was calculated according to the following formula [Equation (12)]:

(12) $$\Delta_{r}G^{0}\left(298K\right)=\sum {\left(\varepsilon_{0}+G_{corr}\right)}_{products}-\sum {\left(\varepsilon_{0}+G_{corr}\right)}_{reactants}$$

where:

- Δ_r_G^0^(298K) is Gibb’s free energy of the reaction, at 298 K (25 °C) and pressure of 1 atmosphere.
- ε_0_ is total electronic energy [Hartree].
- *G_corr_* is thermal free energy [Hartree].

**Figure 5:**
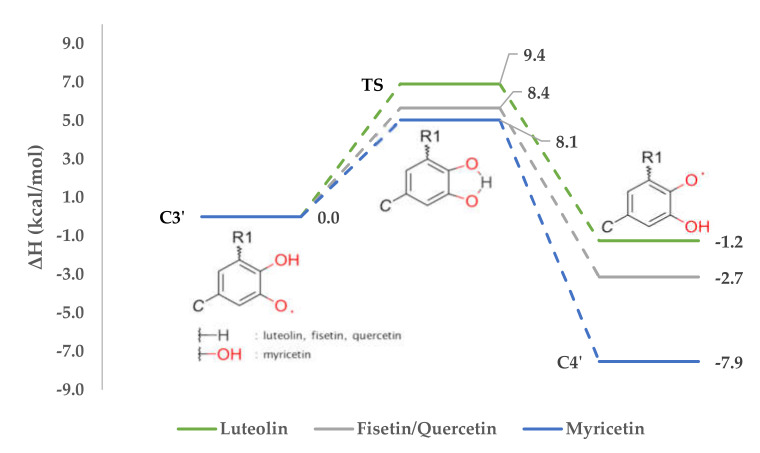
Reactions profile of intramolecular hydrogen swap.

All values necessary for thermochemical calculations and to plot the reactions profile (Figure 5) are presented in Table S3.

According to Table 6, where HAEs are noted, the hydrogen abstraction from fisetin should take place at C3’, while for luteolin, myricetin and quercetin, position C4’ is preferred. In consequence, corresponding radicals will be created. The Gibb’s free energies calculated for these hydrogen swap reactions are respectively −2.5 kcal/mol, 1.9 kcal/mol, 7.5 kcal/mol and 1.9 kcal/mol. The value obtained for fisetin fits with an assumption that the hydrogen from C4’ will likely trade for the free electron at C3’. On the other hand, it can be also stated that the reverse process, the hydrogen swap from C3’ to C4’, is not going to happen.

If hydrogen abstraction takes place at C3’ of luteolin, myricetin or quercetin, an intramolecular swap would happen as well. This allows us to state that despite of investigated compound, if the C4’ radical can be formed by the movement of hydrogen from C3’ or C5’, this will occur, the most stable radical will be formed, and the reaction will be favored thermodynamically. This assumption needs to be tested also on different compounds—e.g., phenolic acids or anthocyanins.

#### 3.5.2. Diradical Formation

To investigate a possible mechanism of the second hydrogen abstraction, the distinctive determinants were calculated for diradical unrelaxed structures, in the same way that radicals were (Table 8).

Bond dissociation enthalpies are usually larger than for the single radical formation, and only luteolin behaves differently. This is correct according to intuition and a knowledge of chemistry—that creating a structure with two unpaired electrons requires more energy. It is not likely that second hydrogen abstraction occurs this way.

Interesting values were achieved for the first step of the ETPT mechanism, because formation of a cation-radical releases nearly 300 kcal/mol. On the other hand, proton dissociation requires nearly 400 kcal/mol; therefore, total average enthalpy required for this process is about 120 kcal/mol. According to the statements made during the investigation into single radicals, the first step shows the thermodynamically preferred pathway. For this reason, the ETPT mechanism is assumed to be responsible for the diradical formation. It is probable that, in studies with explicit water solvent molecules, where hydrogen bonds are involved, the PDE would decrease too.

The first step of the SPLET mechanism has lower enthalpy values than for a single radical, but the second one has higher values. Proton affinities are lower than corresponding values in the single radical formation, and electron transfer energies are quite larger.

#### 3.5.3. o-Hydroquinone Formation

The reorganization of diradicals into o-hydroquinone was determined the same way as the intramolecular hydrogen swap earlier (thermochemical values are presented in Table S4 and shown in Table 9, where o-HFE stands for o-hydroquinone formation enthalpy from diradicals). The results show that the intramolecular reorganization into o-hydroquinone happens since each Gibb’s free energy is negative, especially for fisetin, myricetin C3’–C4’ and quercetin. O-hydroquinone products were experimentally found by Maini et al. [68].

The biggest o-hydroquinone formation enthalpy is also denoted for the three stated compounds—all are below −10 kcal/mol. Relaxation of the molecule decreases the system’s total enthalpy even more. The reorganization enthalpy of luteolin is nearly −35 kcal/mol. Interestingly, the structure, instead of becoming more planar, increases its dihedral angle to 29.6°.

These values are even greater if o-HFE and RE are summed up. In the end, the whole mechanism of catechol moiety was described.

## 4. Conclusions

Within this research, flavonols and flavones were analyzed with the B3LYP/6-31+G(d,p) level of computational chemistry theory, and structure–activity relationship dependencies were proposed. First of all, it was noted that free *p_y_* orbitals play a role when the radical is formed. They contribute to the electron delocalization by resonance and hyperconjugation effects, but only in AC- or BC-ring complexes, depending on the hydrogen abstraction site. The reason for this is the cross-conjugation effect of the carbonyl residue. Nevertheless, there is no possibility that hydrogen density would be exchanged between them in any manner—neither directly, nor indirectly via hydrogen atom exchange. Because of this, most flavonols and flavones adapt geometries, for which AC- and B-rings are coplanar, resulting in conjugation enhancement. It was noticed that an intramolecular hydrogen bonding can be even more important, as flavonoids with two or three B-ring hydroxyl groups close together prefer to detach the hydrogen atom from the B-ring instead of the AC-ring, as suggested by the reorganization enthalpy values. Each additional hydrogen bond guarantees greater reduction of the system’s total enthalpy due to the stabilization effect. Moreover, if the structure involves a dicatechol moiety, it is likely to form the hydroquinone form via the diradical intermediate state, where ETPT plays a role when the diradical is going to be formed. Thermodynamically, the most favored mechanism of action for the first hydrogen abstraction in a polar solvent is a C7 SPLET abstraction. On the other hand HAE, by including reorganization enthalpy correction, we see that C3 for flavonols and C4’ for flavones (especially if a C3’ or C5’ hydroxyl group is present) are most favorable.

## Figures and Tables

**Table 1:** Flavonoid structures investigated in this paper.

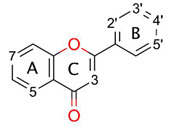								
	Flavonoid	C2’	C3’	C4’	C5’	C3	C5	C7
Flavones	Acacetin			-OCH_3_			-OH	-OH
	Apigenin			-OH			-OH	-OH
	Chrysin						-OH	-OH
	Chrysoeriol		-OCH_3_	-OH			-OH	-OH
	Diosmetin		-OH	-OCH_3_			-OH	-OH
	Genkwanin			-OH			-OH	-OCH_3_
	Luteolin		-OH	-OH			-OH	-OH
Flavonols	Fisetin		-OH	-OH		-OH		-OH
	Galangin					-OH	-OH	-OH
	Kaempferol			-OH		-OH	-OH	-OH
	Morin	-OH		-OH		-OH	-OH	-OH
	Myricetin		-OH	-OH	-OH	-OH	-OH	-OH
	Quercitin		-OH	-OH		-OH	-OH	-OH

**Table 2:** C2–C1’ dihedral angles of molecules, their corresponding radicals and the difference between them (Δ).

	Flavonoid	θ C2–C1’	θ^●^ C2–C1’						
			C2’	C3’	C4’	C5’	C3	C5	C7
Flavones	Acacetin	−13.7						12.9 (Δ = −2.2)	7.9 (Δ = −7.2)
	Apigenin	−15.5			−5.7 (Δ = + 9.8)			−12.5 (Δ = + 3.0)	−8.6 (Δ = −6.9)
	Chrysin	20.6						18.7 (Δ = −1.9)	16.3 (Δ = −4.3)
	Chrysoeriol	−16.9			−3.7 (Δ = + 13.2)			−18.3 (Δ = −1.4)	−4.9 (Δ = + 12.0)
	Diosmetin	16.7		12.1 (Δ = −4.6)				17.6 (Δ = + 0.9)	9.9 (Δ = −6.8)
	Genkwanin	−16.0			−4.5 (Δ = + 11.5)			−14.7 (Δ = + 1.3)	
	Luteolin	15.9		23.5 (Δ = + 7.6)	12.2 (Δ = −3.7)			19.2 (Δ = + 3.3)	8.3 (Δ = −7.6)
Flavonols	Fisetin	8.8		3.3 (Δ = −5.5)	0.0 (Δ = −8.8)		0.3 (Δ = −8.5)		6.2 (Δ = −2.6)
	Galangin	−15.0					0.0 (Δ = + 15.0)	−3.6 (Δ = + 11.4)	−14.2 (Δ = + 0.8)
	Kaempferol	−3.5			0.0 (Δ = + 3.5)		0.0 (Δ = + 3.5)	0.1 (Δ = + 3.6)	−3.4 (Δ = + 0.1)
	Morin	35.7	44.7 (Δ = + 9.0)		32.6 (Δ = −3.1)		0.0 (Δ = −35.7)	34.4 (Δ = −1.3)	33.2 (Δ = −2.5)
	Myricetin	−9.1		−10.1 (Δ = −1.0)	−2.5 (Δ = + 6.6)	−5.3 (Δ = + 3.8)	0.0 (Δ = + 9.1)	−6.3 (Δ = + 2.8)	−7.7 (Δ = + 1.4)
	Quercetin	−8.5		−10.5 (Δ = −2.0)	0.0 (Δ = + 8.5)		0.0 (Δ = + 8.5)	−5.9 (Δ = + 2.6)	−5.7 (Δ = + 2.8)

**Table 3:** Values of bond dissociation enthalpies [kcal/mol].

	Flavonoid	Bond Dissociation Enthalpy						
		C2’	C3’	C4’	C5’	C3	C5	C7
Flavones	Acacetin						102.1	**91.1**
	Apigenin			**89.3**			100.5	91.7
	Chrysin						102.1	**92.8**
	Chrysoeriol			**87.0**			106.7	91.6
	Diosmetin		**87.8**				106.8	92.0
	Genkwanin			**88.8**			102.2	
	Luteolin		88.2	**84.4**			106.1	91.8
Flavonols	Fisetin		**83.4**	85.1		86.7		90.3
	Galangin					**86.9**	98.5	91.7
	Kaempferol			86.7		**85.5**	98.3	90.3
	Morin	93.4		88.8		**84.6**	99.1	91.4
	Myricetin		87.5	**80.7**	84.4	86.1	98.3	91.0
	Quercetin		87.4	**81.4**		85.5	98.0	90.4

The numbers in bold typeface indicate the lowest value for a given compound.

**Table 4:** Values of ionization potentials and proton dissociation enthalpies [kcal/mol].

	Flavonoid	Ionization Potential	Proton Dissociation Enthalpy						
			C2’	C3’	C4’	C5’	C3	C5	C7
Flavones	Acacetin	115.9						16.1	**5.0**
	Apigenin	117.0			**2.2**			13.4	4.6
	Chrysin	120.5						11.4	**2.1**
	Chrysoeriol	113.4			**3.5**			23.2	8.1
	Diosmetin	113.8		**3.9**				22.9	8.1
	Genkwanin	117.2			**1.4**			14.8	
	Luteolin	115.0		3.1	**−0.7**			20.9	6.6
Flavonols	Fisetin	108.9		**4.4**	6.0		7.6		11.3
	Galangin	114.3					**2.4**	14.0	7.2
	Kaempferol	110.2			6.4		**5.2**	18.0	10.0
	Morin	113.3	10.0		5.4		**1.3**	15.7	8.1
	Myricetin	109.0		8.4	**1.5**	5.3	6.9	19.2	11.9
	Quercetin	108.9		8.4	**2.4**		6.5	19.0	11.4

The numbers in bold typeface indicate the lowest value for a given compound.

**Table 5:** Values of proton affinity enthalpies and electron transfer enthalpies [kcal/mol].

	Flavonoid	Proton Affinity Enthalpy							Electron Transfer Enthalpy						
		C2’	C3’	C4’	C5’	C3	C5	C7	C2’	C3’	C4’	C5’	C3	C5	C7
Flavones	Acacetin						45.0	**30.8**						**87.0**	90.2
	Apigenin			31.8			43.3	**31.4**			87.4			**87.1**	90.1
	Chrysin						44.3	**31.0**						**87.7**	91.7
	Chrysoeriol			33.3			45.0	**30.7**			**83.5**			91.6	90.8
	Diosmetin		36.7				45.3	**30.7**		**81.0**				91.4	91.1
	Genkwanin			**31.1**			46.0				87.5			**86.0**	
	Luteolin		35.8	**27.4**			43.8	31.3		**82.3**	86.8			92.1	90.3
Flavonols	Fisetin		30.7	34.1		38.5		**30.0**		82.6	80.8		**78.1**		90.2
	Galangin					35.2	40.8	**30.3**					**81.6**	87.6	91.3
	Kaempferol			32.0		35.5	42.0	**30.1**			84.6		**79.8**	86.2	90.1
	Morin	39.2		32.1		**28.3**	40.6	29.5	**84.0**		86.6		86.2	88.3	91.8
	Myricetin		36.0	**29.1**	30.1	34.9	41.2	29.9		81.3	81.5	84.2	**81.0**	87.0	90.9
	Quercetin		36.7	**27.2**		35.5	41.5	30.0		80.6	84.0		**79.8**	86.4	90.3

The numbers in bold typeface indicate the lowest value for a given compound.

**Table 6:** Values of reorganization enthalpies and hydrogen abstraction enthalpies [kcal/mol].

	Flavonoid	Reorganization Enthalpies							Hydrogen Abstraction Enthalpies						
		C2’	C3’	C4’	C5’	C3	C5	C7	C2’	C3’	C4’	C5’	C3	C5	C7
Flavones	Acacetin						−**8.7**	−5.1						93.5	**86.0**
	Apigenin			−6.7			−**7.1**	−5.0			**82.6**			93.5	86.7
	Chrysin						−**8.6**	−5.6						93.5	**87.2**
	Chrysoeriol			−**7.7**			−2.4	−5.2			**79.3**			104.3	86.5
	Diosmetin		−**7.2**				−2.4	−5.4		**80.6**				104.4	86.6
	Genkwanin			−6.3			−**8.5**				**82.4**			93.7	
	Luteolin		−7.0	−**8.6**			−1.7	−5.0		81.2	**75.9**			104.4	86.8
Flavonols	Fisetin		−7.5	−7.8		−**9.1**		−5.9		**75.9**	77.3		77.6		84.4
	Galangin					−**8.6**	−8.4	−5.8					**78.3**	90.1	85.9
	Kaempferol			−6.9		−**8.7**	−8.5	−5.5			79.8		**76.8**	89.8	84.8
	Morin	−8.4		−6.2		−**12.6**	−9.1	−5.3	85.0		82.6		**72.1**	90.0	86.1
	Myricetin		−6.8	−8.3	−7.2	−**9.4**	−8.5	−6.0		80.7	**72.4**	77.1	76.7	89.8	85.0
	Quercetin		−7.0	−8.0		−**8.8**	−8.7	−5.6		80.4	**73.4**		76.7	89.3	84.9

The numbers in bold typeface indicate the lowest value for a given compound.

**Table 7:** Relative activity of investigated compounds.

Activity	HAT	ETPT	SPLET
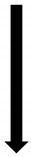	Myricetin	Fisetin, Quercetin	Quercetin
	Quercetin		Luteolin
	Fisetin	Myricetin	Morin
	Luteolin	Kaempferol	Mirycetin
	Morin	Morin	Fisetin
	Kaempferol	Chrysoeriol	Kaempferol
	Galangin	Diosmetin	Galangin
	Chrysoeriol	Galangin	Chrysoeriol, Diosmetin
	Diosmetin	Luteolin	
	Genkanin	Acacetin	Acacetin
	Apigenin	Apigenin	Chrysine
	Accacetin	Genkwanin	Genkwanin
	Chrysine	Chrysine	Apigenin

Flavonoids have been sorted in decreasing order of the enthalpy for the first step of given mechanism.

**Table 8:** Enthalpies of diradical formation mechanisms [kcal/mol].

Flavonoid		HAT	ETPT		SPLET	
		BDE	IP	PDE	PA	ETE
Luteolin		103.6	−282.6	416.1	26.4	107.0
Fisetin		91.6	−287.8	409.2	17.1	104.3
Myricetin	C3’–C4’	90.5	−284.9	405.3	19.7	100.6
	C4’–C5’	83.7		398.5	15.5	98.1
Quercetin		93.8	−285.7	409.4	18.5	105.2

**Table 9:** o-Hydroquinone formation parameters [kcal/mol].

Flavonoid		Δ_r_G^0^	o-HFE	RE	Σ
Luteolin		−5.0	−5.6	−34.4	−40.0
Fisetin		−13.2	−14.4	−15.7	30.1
Myricetin	C3’–C4’	−11.9	−11.9	−14.4	−26.3
Myricetin	C4’–C5’	−7.5	−8.2	−13.8	−22.0
Quercetin		15.1	−14.4	−15.7	−30.1

**Figure A1:**
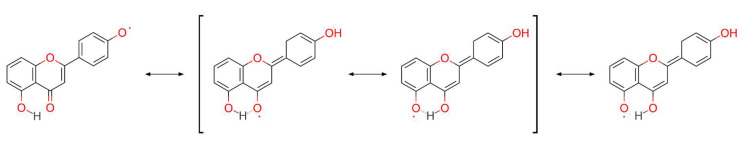
Electron density swap mechanism.

## Supplementary Materials

The following data is available online at https://www.mdpi.com/2076-3921/9/6/461/s1, Figure S1: HOMOs (lower) and LUMOs (upper) visualization with isovalue 0.05, Figure S2: Radicals’ Mulliken spin densities with hydrogen summed into heavy atoms, Figure S3: Enthalpies of flavonoids’ radicals with and without hydrogen bond stabilization (isomer without H-bond is marked *) (a.u.), Table S1: Enthalpies of the flavonoids’ lowest energetic isomer at B3LYP/6-31+G(d,p) level of theory (a.u.), Table S2: Enthalpies of unrelaxed and relaxed radicals of investigated compounds (a.u.), Table S3: Radicals thermochemical values (a.u.), Table S4: Diradicals thermochemical values (a.u.). All geometries are deposited as xyz files in the online repository accessible via link http://dx.doi.org/10.17632/njz3gx3w2d.

## Appendix A. Electron Density Swap

As mentioned earlier, AC- and BC-electron complexes are not in a conjugated system. Thus, electron density is locked in one of them, depending on which the hydroxyl group hydrogen was removed. Looking at Lewis structures (Figure 1) and LUMOs (Figure S1) gives an insight into delocalization, indicating that radicals’ electron density is concentrated on carbonyl oxygen, especially for the C4’ radical. Amić et al. [29] and Heijnen et al. [17] suggested a mechanism of activity, where the hydrogen atom can swap from the C5 position to the carbonyl in exchange for electron density. It was ascertained whether such a transfer from the one complex to another can occur, based on the following mechanism (Figure A1):

Elaboration of the problem was similar to that presented for a catechol moiety. No imaginary frequency was found. For this reason, a different procedure was used—beside the polarizable continuum model, a water molecule was placed near (≈ 2.000Å) to the carbonyl oxygen radical and appropriate hydroxyl group. It was thought that it could serve as a hydrogen bridge between carbonyl oxygen. Based on the calculations, the swap mechanism illustrated in Figure A1 cannot be supported. It seems that electron density cannot be moved between A- and C-rings to achieve further stabilization, at least for the model studied here. This finding emphasizes the importance of the B-ring’s substitution pattern for flavonoids’ antioxidative potential.

## Appendix B. Frontier Molecular Orbitals Theory

### Appendix B.1. Highest Occupied (HOMO) and Lowest Unoccupied (LUMO) Molecular Orbitals

HOMO and LUMO, relatively, are the fundamentals of electron-donating and electron-accepting characteristics in the frontier molecular orbitals theory. The higher the HOMO energy and the smaller the energy gap between HOMO and LUMO, the better the reducing agent is [69]. Since flavonoids operate via hydrogen particle donation, which is coupled with the bound electron transfer, an electron affinity [EA; Equation (B1)] was calculated, and a chemical hardness [η; Equation (B2)] [70] was checked and noted (Table A1). Evaluation of this property was performed according to the following statements:

$$\mathrm{ArOH}\;+{\;e}^{-}\to {\mathrm{ArOH}}^{-}\;$$

(A1) $$\mathrm{EA}\;=\;H\left({\mathrm{ArOH}}^{-}\right)-H\left(e^{-}\right)-H\left(\mathrm{ArOH}\right)$$

(A2) $$\eta \;=\frac{\mathrm{IP}-\mathrm{EA}}{2}$$

**Table A1:** Energies of HOMO, LUMO and chemical hardness of investigated flavonoids [eV].

Flavonoids		E_HOMO_	E_LUMO_	|η|						
				C2’	C3’	C4’	C5’	C3	C5	C7
Flavones	Acacetin	−6.270 *(M)*	−2.217						4.572	4.265
	Apigenin	−6.319 *(L)*	−2.220			4.263			4.512	4.255
	Chrysin	−6.460 *(L)*	−2.320						4.456	4.170
	Chrysoeriol	−6.135 *(M)*	−2.221			4.374			4.625	4.319
	Diosmetin	−6.151 *(M)*	−2.222		4.439				4.624	4.310
	Genkwanin	−6.329 (*L)*	−2.195			4.245			4.565	
	Luteolin	−6.215 (*M*)	−2.240		4.393	4.213			4.565	4.297
Flavonols	Fisetin	−5.924 *(H)*	−2.250		4.413	4.487		4.581		4.398
	Galangin	−6.172 *(M)*	−2.373					4.394	4.514	4.288
	Kaempferol	−5.982 *(H)*	−2.283			4.415		4.491	4.631	4.374
	Morin	−6.141 *(M)*	−2.285	4.505		4.350		4.270	4.534	4.295
	Myricetin	−5.941 *(H)*	−2.315		4.527	4.377	4.399	4.503	4.638	4.396
	Quercetin	−5.934 (*H)*	−2.304		4.544	4.340		4.518	4.647	4.400

E_HOMO_ values do not differ significantly, and the largest can be found for fisetin, kaempferol, myricetin and quercetin. Since chemical hardness tends to be a valuable descriptor of hydrogen donating predisposition [71], flavonols were organized into three classes according to their E_HOMO_ values: low active (L), moderate active (M) and highly active (H). With chemical hardness studies, it can be concluded that flavonoids are hard acids, in the hard and soft acids and bases (HSAB) concept, especially flavonols. Results obtained from these calculations indicate the C7 hydroxyl group as a favorable hydrogen abstraction site. This pattern coincides with IP values from Table 4; similar conclusions were made by Mazzone et al. during hydroxycinnamic acid investigations [72].

A visualization of the results (Figure S1) pinpoints the electron density of HOMO on the B-ring, the C2–C3 double bond and the hydroxyl oxygen atoms. Absence of the hydroxyl groups in the B-ring moves density to the A-ring (e.g., chrysin). The C4’ hydroxyl group is always occupied with a great amount of electron density, even if the adjacent hydroxyl group is not (e.g., myricetin). This is in agreement with the statement that C4’ is the most favored abstraction position. Examination of the LUMO reveals how conjugation between the B-ring and the C2–C3 double bond is formed—one can observe a π-bond swap characteristic for the conjugated system, and creation of C2–C1’ and C3–C4 bonds. Apparently, the electron density arises on the chromone and carbonyl group oxygens. Morin is an exception because B- and C-ring *p_y_* orbitals do not overlap, and thus electrons are retained on them.

### Appendix B.2. Single Occupied Molecular Orbital (SOMO)

Like HOMOs of molecules, SOMOs can be found when radical structures are described. Being occupied by an unpaired electron, these structures are much more reactive than corresponding neutral particles. Simply, to check whether the particle is nucleophilic or electrophilic, one should compare the radical’s E_SOMO_ with the E_HOMO_ and E_LUMO_ of the targeted compound. If the E_SOMO_ is closer to the E_LUMO_, then the radical will act as nucleophile; otherwise its electrophile properties are demonstrated.

Presented in Table A2, E_SOMO_ is similar to the E_HOMO_ of flavonoids they derive from. Since E_SOMO_ is slightly lower than E_HOMO_ for the corresponding molecule, it may suggest they retain electron-donating properties, and would willingly detach another hydrogen to scavenge another radical. DFT results indicate each SOMO was occupied exactly by one electron.

**Table A2:** Energies of SOMOs of investigated flavonoids’ radicals [eV].

Flavonoids		E_SOMO_						
		C2’	C3’	C4’	C5’	C3	C5	C7
Flavones	Acacetin						−6.367	−6.502
	Apigenin			−6.570			−6.456	−6.586
	Chrysin						−6.699	−6.925
	Chrysoeriol			−6.470			−6.205	−6.320
	Diosmetin		−6.471				−6.216	−6.319
	Genkwanin			−6.594			−6.429	
	Luteolin		−6.494	−6.557			−6.343	−6.440
Flavonols	Fisetin		−6.329	−6.290		−6.212		−6.195
	Galangin					−6.463	−6.429	−6.573
	Kaempferol			−6.356		−6.230	−6.176	−6.336
	Morin	−6.237		−6.489		−6.400	−6.305	−6.445
	Myricetin	−6.202	−6.246	−6.265	−6.268		−6.102	−6.258
	Quercetin	−6.189	−6.224	−6.292			−6.122	−6.270

According to the obtained results (Table A3), it is clear that luteolin (flavone) SOMO orbitals are quasi-degenerated. The energy gap between them is much lower than for flavonols, where it reaches approximately 0.7–1.2 eV. Moreover, their energies are even lower than for the molecule’s HOMO or the radical’s SOMO, which the diradical originates from. As a result, SOMO with energy −6.704 eV conforms to other examined diradicals’ lowest energetic SOMO.

**Table A3:** Energies of SOMOs of investigated flavonoids’ diradicals [eV].

Luteolin	Fisetin	Myricetin		Quercetin
*C3’-C4’*	*C3’-C4’*	*C3’-C4’*	*C4’-C5’*	*C4’-C5’*
−6.704	−5.989	−6.049	−6.003	−6.032
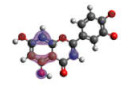
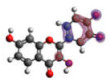
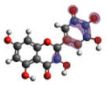
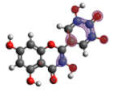
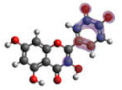
−6.893	−7.184	−6.721	−6.757	−6.866
