# Supplementary material for: Flavones’ and Flavonols’ Antiradical Structure–Activity Relationship—A Quantum Chemical Study
Source: Antioxidants (Basel). 2020 May 27;9(6):461. doi: 10.3390/antiox9060461 (PMC7346117; doi:10.3390/antiox9060461)
Supplement: Supplementary file 1 [file antioxidants-09-00461-s001.pdf]

antioxidants

MDPI

Supplementary Materials

Figures

Acacetin	Apigenin	Chrysoeriol

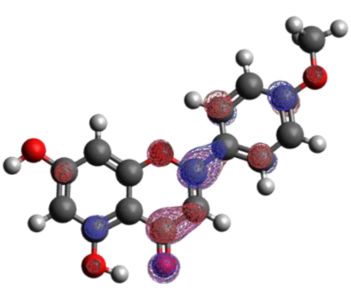
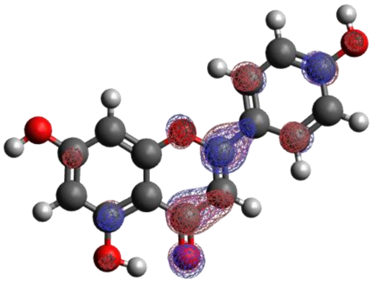
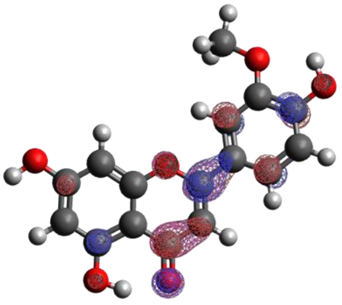
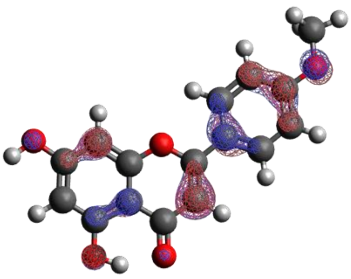
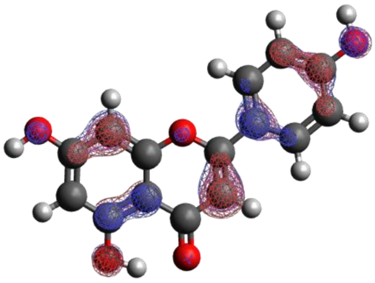
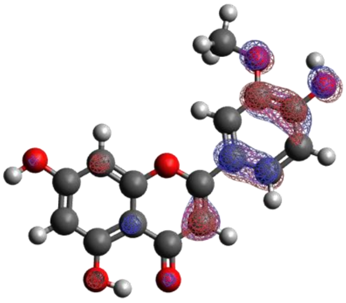

Chrysine	Diosmetin	Fisetin

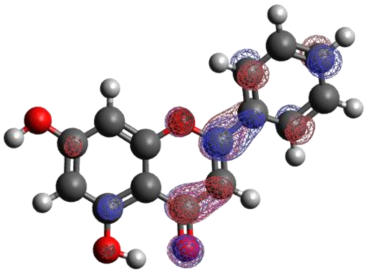
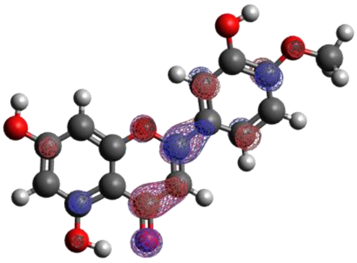
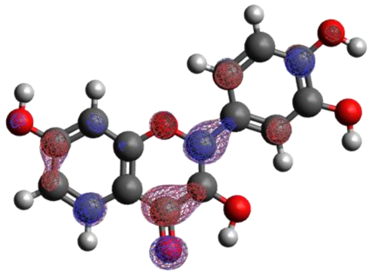
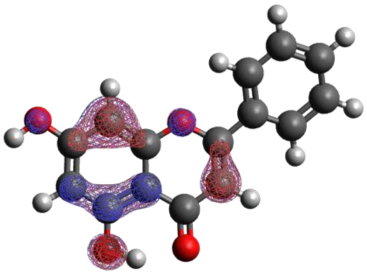
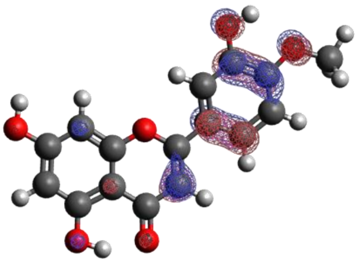
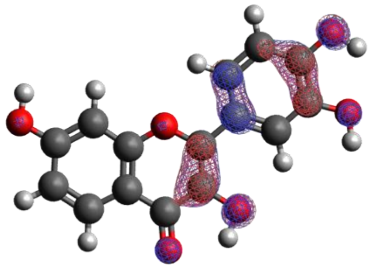

Genkwanin	Galangin	Kaempferol

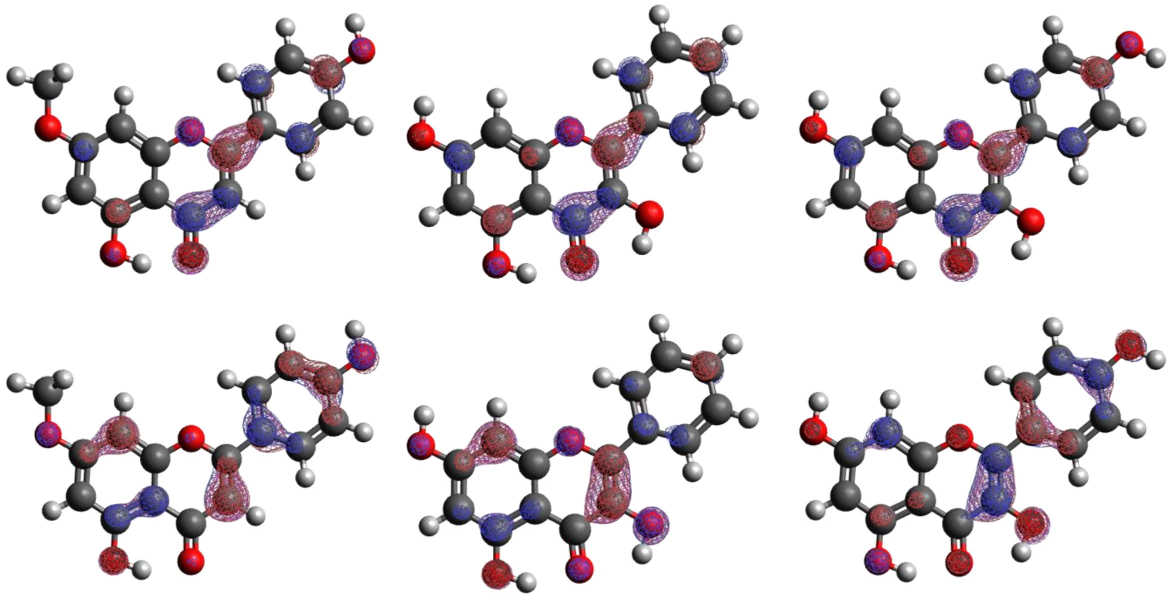

Luteolin **Morin** **Myricetin**

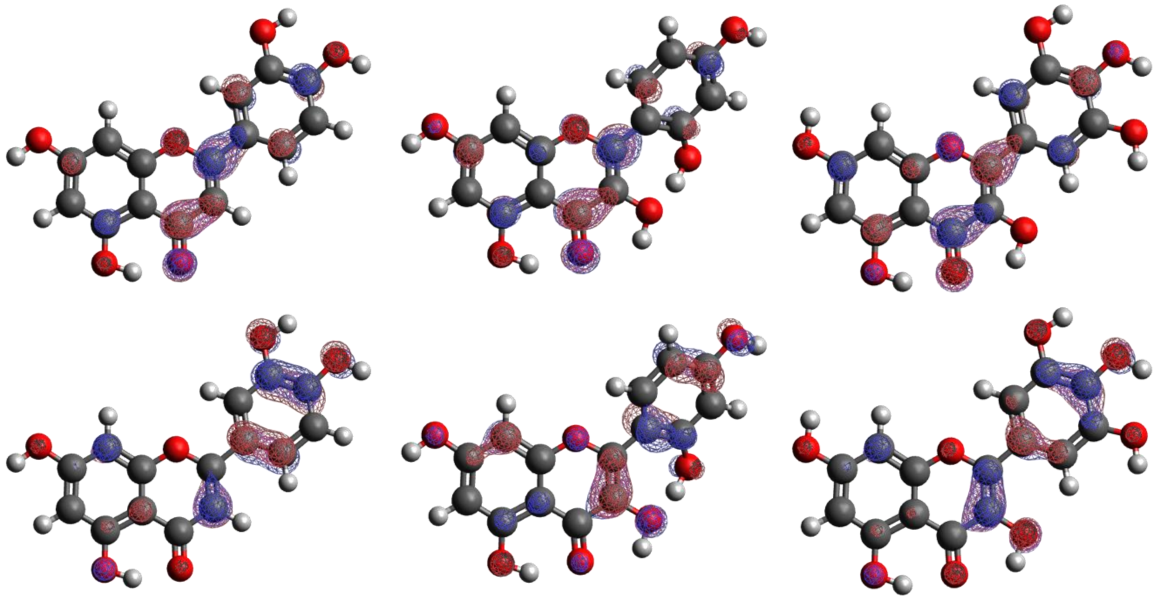

Quercetin

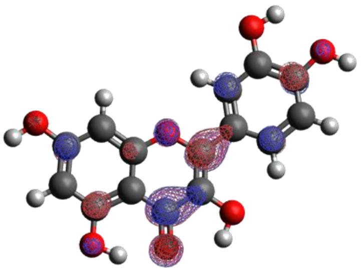

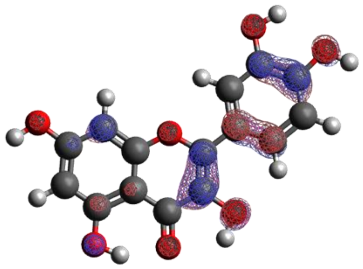

Figure 1. HOMOs (lower) and LUMOs (upper) visualization with isovalue 0.05

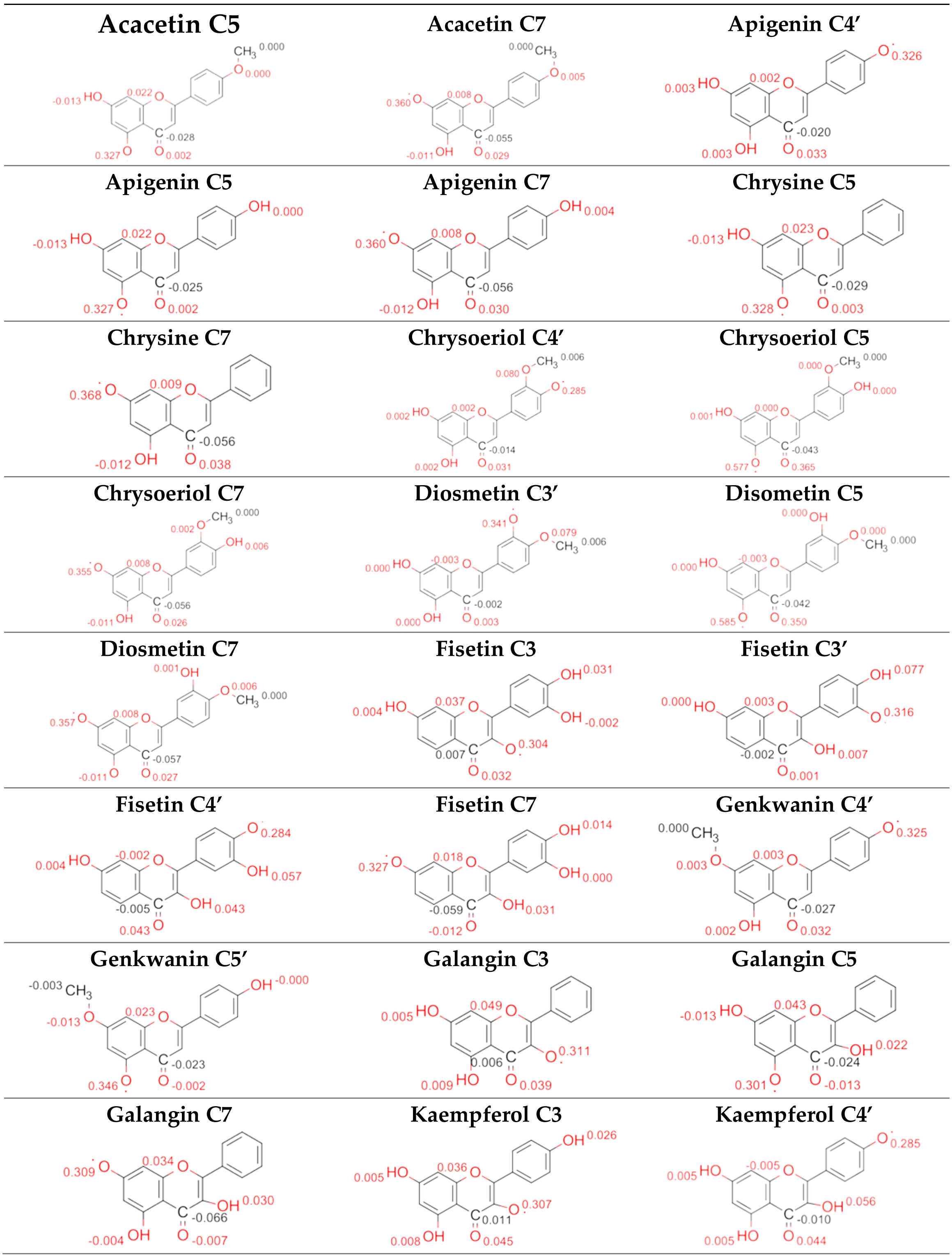

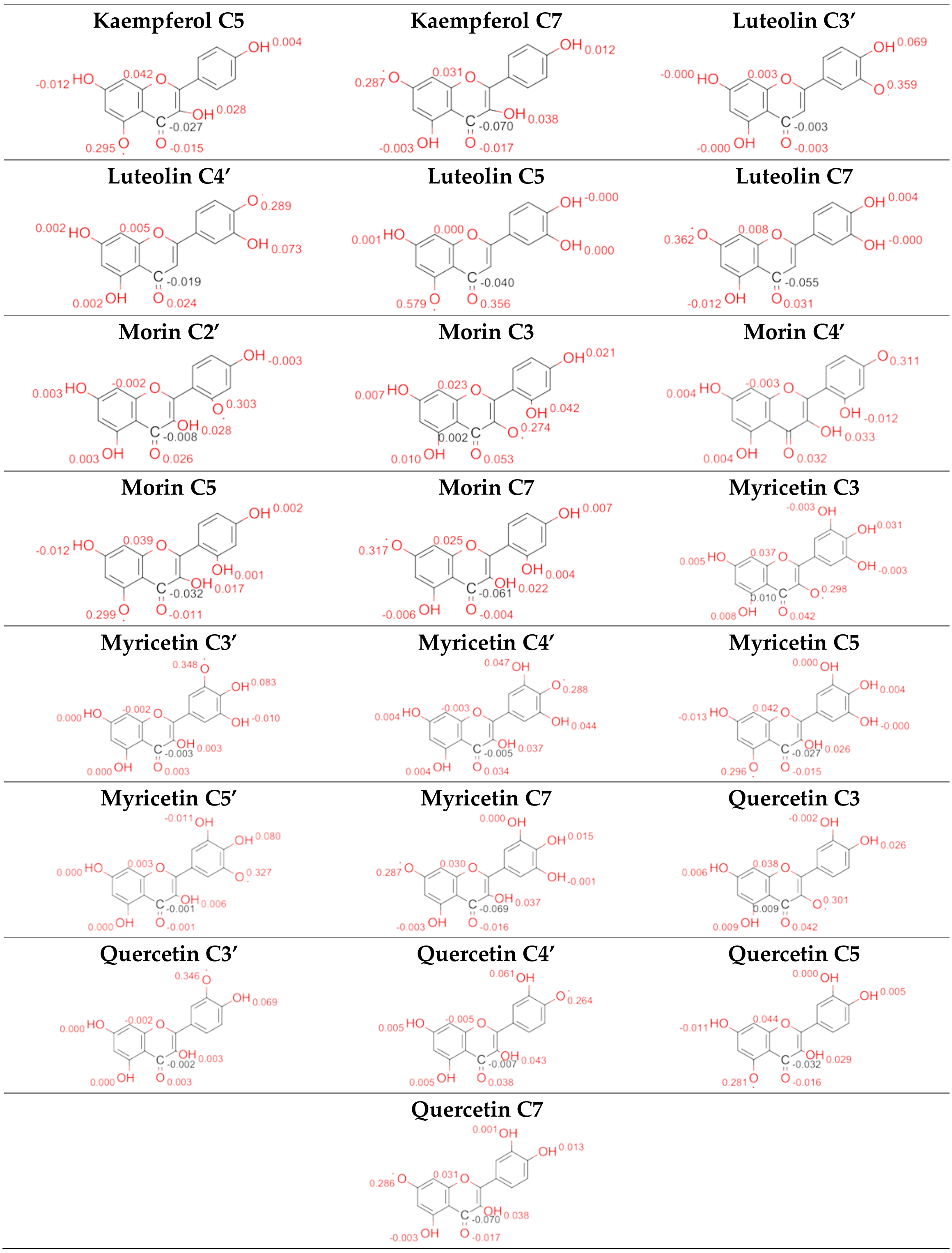

Figure 2. Radicals′ Mulliken spin densities with hydrogen summed into heavy atoms

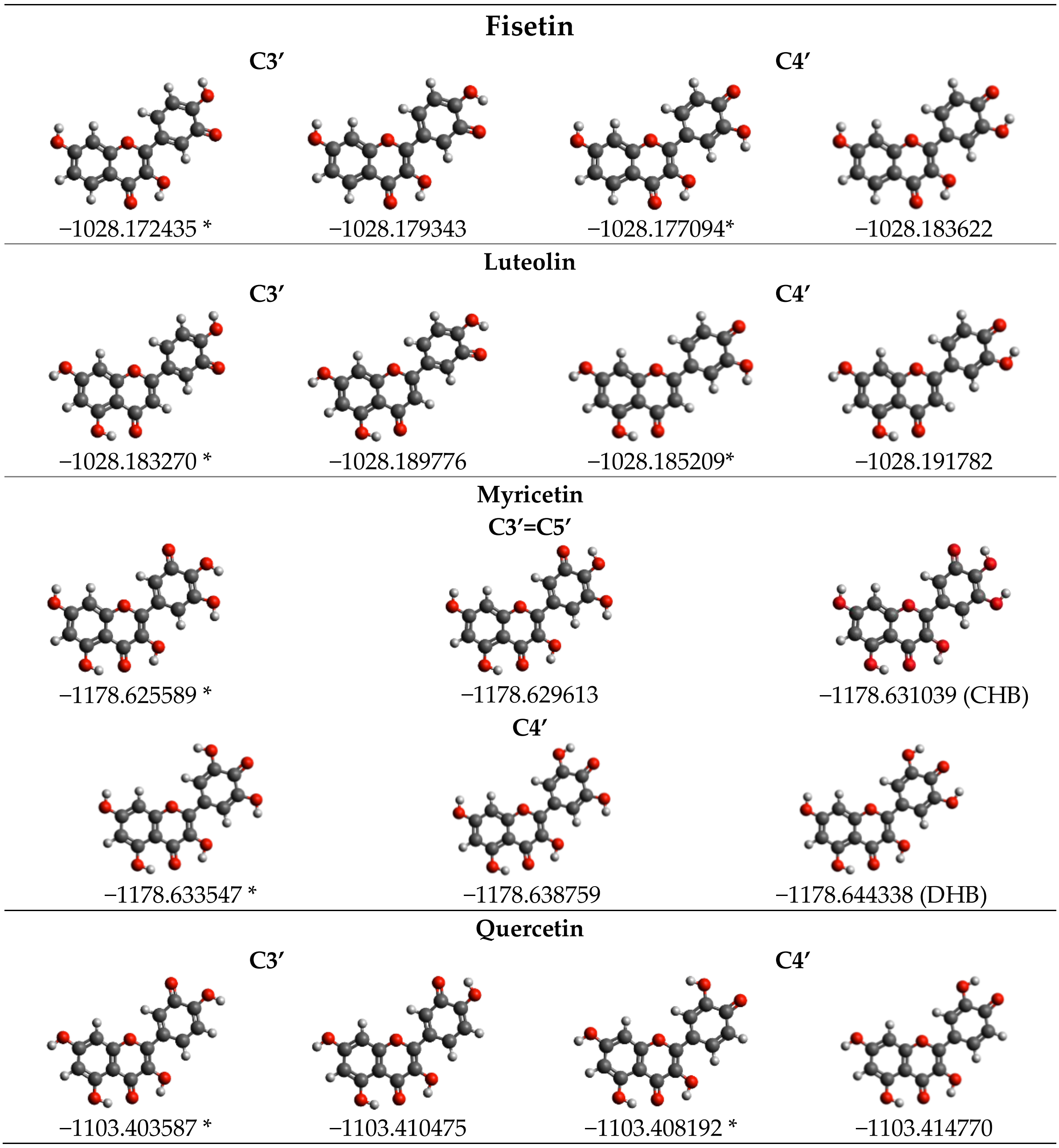

Figure 3. Enthalpies of flavonoids′ radicals with and without hydrogen bond stabilization (isomer without H−bond is marked *) [a.u.]

Tables

Table 1. Enthalpies of the flavonoids' lowest energetic isomer at B3LYP/6–31+G(d,p) level of theory [a.u.]

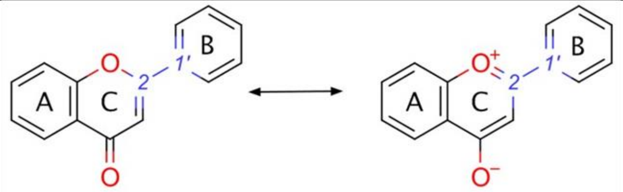

	Flavonoid	Enthalpy
Flavones	Acacetin	−992.861147
	Apigenin	−953.589415
	Chrysin	−878.363148
	Chrysoeriol	−1068.084598
	Diosmetin	−1068.084312
	Genkwanin	−992.861115
	Luteolin	−1028.811831
Flavonols	Fisetin	−1028.799467
	Galangin	−953.582425
	Kaempferol	−1028.808400
	Morin	−1104.033327
	Myricetin	−1179.253341
	Quercetin	−1104.030864

Table 2. Enthalpies of unrelaxed and relaxed radicals of investigated compounds [a.u.]

	Flavonoids		Unrelaxed radical	Relaxed radical
Flavones	Acacetin	C5	−992,199222	−992,213026
		C7	−992,216822	−992,224983
	Apigenin	C4′	−952,947947	−952,958621
		C5	−952,930000	−952,941269
		C7	−952,944139	−952,952039
	Chrysin	C5	−877,701313	−877,715043
		C7	−877,716141	−877,725026
	Chrysoeriol	C4′	−1067,446832	−1067,459122
		C5	−1067,415336	−1067,419226
		C7	−1067,439373	−1067,447632
	Diosmetin	C3′	−1067,445198	−1067,456637
		C5	−1067,414907	−1067,418746
		C7	−1067,438585	−1067,447177
	Genkwanin	C4′	−992,220493	−992,230596
		C5	−992,199106	−992,212595
	Luteolin	C3′	−1028,172077	−1028,183270
		C4′	−1028,178156	−1028,191782
		C5	−1028,143630	−1028,146283
		C7	−1028,166412	−1028,174353
Flavonols	Fisetin	C3′	−1028,167323	−1028,179343
		C4′	−1028,164704	−1028,177094
		C3	−1028,162134	−1028,176690
		C7	−1028,156325	−1028,165763
	Galangin	C3	−952,944720	−952,958463

	C5	−952,926275	−952,939686
	C7	−952,937061	−952,946355
Kaempferol	C4′	−1028,171031	−1028,182027
	C3	−1028,173008	−1028,186897
	C5	−1028,152508	−1028,166096
	C7	−1028,165319	−1028,174020
Morin	C2′	−1103,385259	−1103,398629
	C4′	−1103,392680	−1103,402521
	C3	−1103,399249	−1103,419264
	C5	−1103,376267	−1103,390761
	C7	−1103,388420	−1103,396868
Myricetin	C3′	−1178,614708	−1178,625589
	C4′	−1178,625608	−1178,638759
	C5′	−1178,619670	−1178,631215
	C3	−1178,616992	−1178,631960
	C5	−1178,597496	−1178,611019
	C7	−1178,609123	−1178,618703
Quercetin	C3′	−1103,392412	−1103,403587
	C4′	−1103,401993	−1103,414770
	C3	−1103,395481	−1103,409514
	C5	−1103,375549	−1103,389360
	C7	−1103,387556	−1103,396441

Table 3. Radicals thermochemical values [a.u.]

Flavonoids	Radical	$\varepsilon_0 + H_{corr}$	$\varepsilon_0 + G_{corr}$
Luteolin	C3′	−1028.190	−1028.252
	TS	−1028.179	−1028.240
	C4′	−1028.192	−1028.255
Fisetin	C3′	−1028.179	−1028.243
	TS	−1028.170	−1028.232
	C4′	−1028.184	−1028.247
Myricetin	C3′=C5′	−1178.631	−1178.699
	TS	−1178.623	−1178.691
	C4′	−1178.643	−1178.711
Quercetin	C3′	−1103.410	−1103.477
	TS	−1103.401	−1103.466
	C4′	−1103.415	−1103.480

Table 4. Diradicals thermochemical values [a.u.] [ii.]

Flavonoids		$\varepsilon_0 + H_{corr}$ [i]		$\varepsilon_0 + G_{corr}$
Luteolin		−1027.514		−1027.574
		−1027.523	−1027.578 *(r)*	−1027.582
Fisetin		−1027.522		−1027.583
		−1027.545	−1027.570 *(r)*	−1027.604
Myricetin	*C3′−C4′*	−1177.982		−1178.046
		−1178.001	−1178.024 *(r)*	−1178.065
	C4′−C5′	−1177.993		−1178.057
		−1178.006	−1178.028 *(r)*	−1178.069
Quercetin		−1102.753		−1102.815
		−1102.776	−1102.801 *(r)*	−1102.839

[i] *(r)* marks for the relaxed forms of o−hydroquinone.

[ii] Underlined numbers stand for o−hydroquinone unrelaxed form.

© 2020 by the authors. Submitted for possible open access publication under the terms and conditions of the Creative Commons Attribution (CC BY) license (http://creativecommons.org/licenses/by/4.0/).
